# How can medical schools create the general practice workforce? An international perspective

**DOI:** 10.15694/mep.2020.000267.1

**Published:** 2020-11-27

**Authors:** Davinder Sandhu, Andrew Blythe, Vijay Nayar

**Affiliations:** 1 formerly Royal College of Surgeons in Ireland - Bahrain; 2 University of Bristol; 3 Health Education England

**Keywords:** Primary care, family medicine, undergraduate curriculum, GP workforce, international.

## Abstract

This article was migrated. The article was marked as recommended.

Medical schools are responsible for producing the future medical workforce that is fit for purpose to meet the needs of patients. The burden of care largely falls upon primary care, a specialty which has been under-doctored, under-resourced, culturally not valued, and whose status has been subordinate to secondary care. In addition, the demographic changes of increasing longevity and chronic diseases, have increased the demand for healthcare at a time when governments’ finances are stretched more than ever. The poor state of recruitment to the primary care workforce is a global phenomenon as evidenced by similar issues in North America, UK and the Middle East including Turkey. This contradicts the fact that the strength of primary care has a direct effect on the health of the nation.

This paper presents an international perspective of why primary care is important, the reasons why new graduates shy away from a career in primary care, and what steps medical schools, residency programmes and governments should undertake to encourage medical students to make primary care a preferred career choice.

## Introduction

The healthcare needs of people are evolving all the time, and particularly rapidly during the COVID-19 pandemic. In addition, increasing longevity, associated with a growing burden of non-communicable diseases (NCDs) and budgetary constraints have created huge challenges for all global health systems including public health and primary care ( Marmot, 2005). The task of dealing with these demands falls largely to primary care workers who are in the front line of healthcare for patients residing in the community. The General Practitioner (GP) workforce has been under scrutiny; challenges have been identified in both recruitment and retention. The World Health Organisation (WHO) defines the social accountability of medical schools as “the obligation to direct their education, research and service activities towards addressing the priority health concerns of the community, region and the nation that they have a mandate to serve”. Thus a key function of medical schools is to supply the primary care workforce which is essential for the health needs of the community ( Jason and Douglas, 2015).

## Why is primary care important?

Previous studies have demonstrated that high quality primary care is cost effective, reduces health inequalities, and improves patient outcomes ( Starfield, 1994; Macinko, Starfield and Shi, 2007). Well established primary care systems also buffer the health effects of deprivation and at lower cost than healthcare systems that rely more extensively on secondary and tertiary care ( Starfield, Shi and Macinko, 2003).

Primary care is a key component in reducing a country’s overall burden of disease. This has been well documented in the 18 wealthy Organisation for Economic Cooperation and Development (OECD) countries over three decades ( Macinko, Starfield and Shi, 2003). The Canadian National Population Health Survey and the Canadian Institute for Health Information public health study, demonstrated that a higher supply of specialists to general practitioners ratio is correlated with worse health outcomes, while the converse has better health outcomes ( Piérard, 2009).

## Why is there poor uptake of general practice from graduates of medical schools?

In the report ‘By Choice and Not by Chance’, Wass, Gregory and Petty-Saphon (2016) set out the factors that influence medical students in the UK when they start to explore career options. These factors reflect hidden values and expectations firmly entrenched within the medical profession. There are three significant deeply seated issues affecting students; “tribalism, negativism and finance”.

### Tribalism

Unfortunately, professional tribalism drives the belief that primary care is of a minor status compared to hospital practice. Primary care is then seen as a forced necessity to relieve the pressure in overcrowded urban teaching hospitals. The lesser status afforded to GPs by secondary care deters medical students from contemplating primary care as a career.

### Negativism

Students also perceive that primary care fails to offer academic challenges that they may aspire to. They assume that specialist services can and should only be delivered in secondary care. In the community primary care provides the initial healthcare and subsequent follow up. The local GP is the generalist, and the role of the secondary care specialist services is to assist the GP in a partnership to manage the needs of the patient. There is also the perception that GPs in the UK and USA are over-burdened by the electronic medical record (EMR). A medical student sees a consultation more focussed on the screen than on the patient, and contrasts this with how little time specialists in hospitals spend on EMR which further deters them away from primary care ( Ostrander, 2018).

### Finance

This can be broken down to the cost of delivering student teaching in general practice and the impact of student debt and remuneration of GPs.

### Cost of delivering teaching in primary care

There is the lack of equity of recompense for undergraduate education teaching across the diverse healthcare settings ( Wass, Gregory and Petty-Saphon, 2016). Although the UK has a national rate for undergraduate teaching in secondary care, there is not a national rate for undergraduate teaching in primary care, and on average the undergraduate tariff for primary care is well below that of secondary care. Delivering more teaching in primary care requires more GP teachers. In the UK in 2014, only 4,392 practices (40%) taught medical students ( Derbyshire *et al*., 2014). This is a small increase from the 3,900 practices (35%) who taught 12 years earlier ( Derbyshire *et al*., 2014). Further expansion of the teaching capacity in primary care will require investment in GP premises to create space, and a new tariff that makes it viable for GPs to teach. In the UK, such negotiations between the Society for Academic Primary Care (which represents Heads of Teaching of Primary Care at UK medical schools), and the Department of Health and Social Care will need to have constructive dialogue to address these issues ( Blythe, 2018). There is some ground for optimism on this score because in 2020 the Department of Health in England announced that the primary care teaching tariff in all parts of the country would be increased to that of the highest tariff, although that is still below the secondary care tariff.

### Student debt and Remuneration of Primary Care physicians

Accumulated student debt is a major problem for USA medical graduates ( Nykiel, 2019) and in the UK the dramatic rise in university fees over the last decade is creating a similar problem. In the USA this debt eventually influences the choice of specialty, with new graduates invariably selecting higher paying specialties. Canadian medical students do not incur these high education debts in comparison to USA students due to government subsidies and they are therefore more likely to be attracted to general practice ( Whitcomb and Desgroseilliers, 1992). The USA and Canadian primary-care physicians pay scales are similar, but the disproportionate difference in income between primary care physicians and specialists is smaller in Canada (30%) compared to the USA (50% - 100%). Inevitably, students especially with large debts are attracted to hospital specialties ( Hayward, Kravitz and Shapiro, 1991) and therefore the USA struggles to recruit and retain GPs ( Whitcomb and Desgroseilliers, 1992).

Canadian students however, view the relative lower income of family physicians less of a discouragement to be a family care physician, as they feel GPs are valued and enjoy a high status in society.

## How is the Primary Care workforce being developed internationally?

### United Kingdom

In the UK the number of GP consultations continues to increase; it now stands at around 328 million consultations per year ( Hobbs *et al.*, 2016). Yet the number of GPs per head of population in the UK is falling, and GPs make up a smaller percentage of the doctor workforce than they did 20 years ago. In 2009 the number of GPs per 100,000 head of population was 67, reducing to 60 in 2018 and in the mid-1990s, GPs formed 34% of the total doctor workforce; now they represent just 26% of the total doctor workforce ( Palmer, 2019). Gender and early retirement also play a part. Of the GPs in training 65% are women; it is important that this major group are supported during their family break and not lost to the profession ( Bienkowska-Gibbs *et al*. 2015). In a recent survey by the University of Warwick (2019), the number of GPs planning to leave or retire from practice in the next five years had risen to 42.1% of GPs compared to 31.8% of those surveyed in the same region in 2014. Meanwhile general practice’s share of UK NHS funding has edged up from a historical low of 7.4% in 2014/15 ( Baird *et al.*, 2016) to a projected budget of only 10.1% of the total NHS budget by 2020/21 ( General Practice Forward View, 2016) despite the fact that 90% of all patient contacts take place in primary care ( Hobbs *et al*., 2016).

In order to rebalance the UK NHS workforce, medical schools in the UK have been told that 50% of their graduates need to enter general practice ( Dept. of Health, 2013). In surveys conducted across the UK from 2002 to 2009, only 20-22% of doctors in their first year after qualification put general practice as their unequivocal first choice for their eventual career ( Lambert and Goldacre, 2011). In a further survey the figure was 19% ( UK Foundation Programme, 2019). If doctors who express any preference for general practice are included, then the 50% target is reached, but many of these doctors end up in other specialities ( Lambert and Goldacre, 2011). In 2019, 31.6% of foundation doctors (interns) were appointed to a GP training programme ( UK Foundation Programme, 2019).

In order to cultivate students’ enthusiasm for this career, general practice must be central to the undergraduate curriculum. Unfortunately, until very recently the proportion of the undergraduate medical curriculum devoted to general practice was in decline. From 2002 to 2015 the amount of time that students spent in general practice during their last three years at medical school fell by two weeks, to around 13% of the curriculum ( Harding *et al.*, 2015). Undergraduate exposure to general practice positively influences future career choice across all medical schools ( Alberti *et al*., 2017). Cambridge medical school has re-designed its curriculum, putting much more emphasis on general practice, and has seen an increase in its number of graduates who enter general practice training from 7% to 22% ( Millet, 2017).

The capacity of medical schools to teach general practice is also hampered, by the fact that the number of medical schools with departments of general practice has halved since 2002 ( Harding *et al*., 2015). This has reduced the opportunities for students to encounter role models or learn about the intellectual foundations of the discipline. Academic primary care could be fostered by encouraging more students to apply for academic foundation posts which include research or teaching in primary care.

### United States (USA)

The USA primary care workforce is inadequate to meet the needs of their population ( Association of American Medical Colleges, 2012). This leaves the nation’s most vulnerable populations - the uninsured, low-income groups, those in rural or inner-city areas - with limited care ( Jones and Berk, 2012). Healthcare education need to support a more diverse medical student population that better represents the community they serve, and one that is more likely to choose primary care careers and practise in underserved areas. According to the Kaiser Family Foundation, 56 percent of USA patient visits are to primary care clinicians, but only 37 percent of physicians practice primary care medicine. Residency training positions must increase steeply in family medicine. In 2008, 35% of the USA physician workforce were in family medicine but that rate is reducing ( Kozakowski *et al*., 2017). Historically, there was declining interest amongst graduates of USA medical schools’ careers in primary care medicine. Between 1986 and 1991 residents matched to family medicine decreased by 19% ( National resident matching programme, 1986, 1991). Such alarming data when extrapolated suggests that there could be a 25% shortfall in primary-care physicians ( National resident matching programme, 1991; Politzer, 1991; Colwill, 1992; Petersdorf, 1992). The Carnegie Foundation report, Educating Physicians ( Cooke, Irby and O’Brien, 2010), highlighted three negative parameters to deliver efficient and effective care namely “high tuition fees, student debt, and low numbers in the primary care physician workforce”.

The USA primary care crisis will feel the strain as a result of the Affordable Care Act and others that may follow, as millions more Americans enter a health care system that is ill equipped to look after them and therefore the mis-match between the requirements of the public and output from the residency programmes needs to be addressed.

The Association of American Medical Colleges (AAMC) and USA medical schools in their report (2020) have recognized this potential workforce crisis and have committed to increasing the number of graduating medical students by 15 to 30% and aim to have 25% of medical school output enter family medicine by 2030. This led to a serious rethink in American public health policy. A key strand of the policy consisted of expanding medical school numbers and family medicine placements. The number of medical schools in the USA by 2020 have expanded to a total of 179, including 36 osteopathic schools. Since 2003, when the acceptance rate for family medicine was low at 76.2% there has been rising uptake for positions in family medicine. A record high acceptance rate of 96.7% for family medicine was achieved in 2018 with only 11 positions unfilled post-SOAP (NRMP Supplemental Offer and Acceptance Program®) and 33 unfilled in 2019 ( American Academy of Family Physicians 2020 match results, 2020). In the 2020 match Family Medicine offered 557 more positions than in 2019 at 4,685 places. This was 13.79 % of overall residency programs, out of which 4,335 students/graduates were accepted, the most in its history and 487 more than in 2019. This represents 12.6% of all USA applicants, but half of the goal of at least 25% by 2030 ( American Academy of Family Physicians 2020 match results, 2020).

The 350 family medicine positions unfilled after the first round of the 2020 NRMP Match are anticipated to be filled during the SOAP process. Therefore, while during the past decade there are increasing numbers of positions offered and filled in family medicine, the pace needs to increase significantly to reach the national target of 25% of all USA medical schools and graduates pursuing a career in family medicine ( American Academy of Family Physicians 2020 match results, 2020).

The crisis will continue unless the increasing imbalance of family medicine to hospital specialists is addressed, along with efforts to increase the number of medical school graduates. The 2010 Council of Graduate Medical Education (COGME) report, “Advancing Primary Care” recommended “that policies should be implemented that raise the percentage of primary care physicians in the physician workforce to a minimum of 40%” ( Kozakowski *et al*., 2017). The USA in some places is implementing shortened training time through fast tracking students “who are ensured of preferential admission to generalist residency programs” ( Kozakowski *et al*., 2017).

Further analysis of the annual report from the 2016 NRMP match shows that the production of at least 30 or more graduates entering family medicine was in only 10 of the allopathic medical schools ( Kozakowski *et al*., 2017). Eighty per cent of the family medicine graduates came from 54% of the schools (73/136), while extraordinarily from 8 schools, only one graduate entered family medicine. The leader was the University of Minnesota Medical School which produced 45 graduates ( Kozakowski *et al*., 2017).

### Canada

In Canada, where primary care has been prioritised and received investment, there is strong uptake by graduates, and the Canadian healthcare system has a balanced workforce between the primary and secondary care of approximately 50% each ( Whitcomb and Desgroseilliers, 1992) though the situation is fluid as demands for healthcare rise ( Commission on the future of health care in Canada, 2002).

Nearly 50% of Canadian medical school graduates also choose a specialty that would predispose them to a career in family medicine. This success in contrast to the USA is based on many factors and summarised by Whitcomb and Desgroseilliers (1992):

• The Royal College of Physicians and Surgeons of Canada (RCPSC) represents the primary care workforce as well as the other specialties. The medical profession therefore has a joint voice, whereas in the USA each speciality has its own organisations, which operate in an autonomous manner.
• The financing policies adopted by the provincial governments means that graduate medical education is subsidised. Family medicine departments in medical universities fund residency programs. This is linked to governance and workforce planning by the provincial governments to ensure, that the community needs of primary care are met in their areas.
• Therefore, unlike the USA and other countries, the new graduates are not in serious debt and this can give students the freedom to determine their choice of specialty ( Hayward, Kravitz and Shapiro, 1991).
• In the state of Quebec which has high quality outcomes for community patients, family physicians have to be licensed in their field to practice through completing formal training in family medicine. Furthermore, hospital Consultants are dissuaded from offering primary care services as the latter have their own specialists.
• Additionally, the national, provincial governments and the RCPSC have ensured that family medicine is valued with a high status. The success of this policy can be measured by the fact that residency programmes in family medicine are oversubscribed.

The above has established why Canada is achieving better health outcomes than many OECD countries as well as their high generalist to specialist physician ratio ( Macinko, Starfield and Shi, 2003; Piérard, 2009). The success has continued despite multiple systems of private health care delivery, through strong government and professional leadership ( Hutchison, 2011).

### Middle East

The impact of primary care on population health was the trigger for establishing primary care health centres in the Middle East. Primary care residencies in the Middle East are changing from a traditional curriculum, theory-based to a modern practice-based curriculum, as demonstrated by the Kuwait family residency programme (KFRP), (2019). KFRP collaborates with the Royal College of General Practitioners, and is now a 5-year programme. Initially only a few countries introduced primary care to their medical school curricula. Those that were pioneers in this were Lebanon, Turkey, Bahrain, Jordan and Iraq ( Abyad *et al*., 2007). However, implementation of the structure and delivery of primary care is variable and there can be a lack of coordination between services. The main difficulty was not enough trained indigenous primary care doctors and allied health care workforce. In the past, the total healthcare workforce in primary care including allied health workers in primary care accounted for around 10% of the healthcare workforce. The remainder were recruited from abroad ( Abyad *et al*., 2007).

Lebanon has government clinics and non-governmental organizations (NGOs) providing primary care services. The residency program in Lebanon at the American University of Beirut started in 1979. In Bahrain, in 1978 a four-year training programme with affiliation to the Irish College of General Practitioners and the Royal College of Surgeons in Ireland was established in 1978, and consolidated in 1996 ( Abyad *et al*., 2007). The KFRP started in 1987 that has led to an increase in the number of residents entering family medicine, from 13 (2%) in 1987 to 252 (26%) in 2002 (KFRP, 2019). Qatar and the United Arab Emirates established their primary care programme in 1994 in response to the need for improving the quality of healthcare in the community and to attract GPs to their countries. Jordan had a four-year residency program family medicine in 1981 and the first board examination was established in 1986 ( Abyad *et al.*, 2007). Libya with its 1177 healthcare centres is still struggling with the concept of primary care medicine (Hinish, 2017). Management of chronic disease and education of self-care is limited. However, immunization in excess of 95% against tuberculosis and measles have been a success, with morbidity and mortality now being in mainly non-communicable diseases.

### Turkey

Turkey, as part of Europe, has also faced challenges in their primary care health system. This includes reforms such as decentralisation, provider deregulation, performance-based payments and focus on consumer choice( Öcek *et al.*, 2014). In 1961, the Turkish Ministry of Health established primary care health centres but Turkey only accepted primary-care as a medical specialty in 1984 ( Abyad *et al*., 2007). It took until 2003, before family medicine was introduced as a primary care model responding to the health needs of the population with a promise of long term government support (Turkey Health System Review, 2011). Turkey has grown its primary care workforce ( Tanriover *et al*., 2014) and, according to 2016 data, Turkey has 86,332 doctors and of which 37,173 (43%) are general practitioners and 8615 in a residency program ( Turkish Health Ministry Reports, 2016). This steady increase is still inadequate for a population of approximately 73 million ( Öcek *et al.*, 2014).

There are 73 state and 27 private medical schools in Turkey which between them produce approximately 11,700 medical graduates each year (Sputnik news, 2018). Only 79 of these medical schools have departments of family medicine, and therefore in 20% medical schools, students do not encounter family medicine as a recognized discipline which will have an impact on career choice.

As in other countries the demand for primary care is rising. In 2002 the volume of primary care services (number of visits) was 74.8 million, by 2011 it had reached 244.3 million, while the number of primary care physicians increased from 17,800 to 22,073 in the same period ( Öcek *et al.*, 2014). This supports the Inverse Care Law in healthcare that the the needs of the population are inversely related to the resources invested ( Hart, 1971).

The primary health care system in Turkey mainly consists of two parts: GPs in Emergency Departments who bolster emergency services, and GPs in Family Health Centres who are authorized to work as family physicians without specialization, within the scope of the Health Transformation Program. Medical graduates are unable to obtain in-depth training for Family Medicine (FM) due to the inadequate number of FM specialists. In addition, some of the GPs have to spend compulsory time in Emergency Medicine. In a qualitative study on the reasons that influence Turkish medical students’ decision to become family physicians, the low status of family physicians was cited as a major factor ( Öcek *et al.*, 2014). FM was perceived as a career failure. There was also a perception that FM is not a speciality suitable for doing research. Family physicians were perceived as service providers who ‘have to obey patients demands for prescription refills’, rather than being allowed to practise medicine within their full scope.

Another criticism was the lack of postgraduate training and continuous professional development; the stigma associated with this meant that the patients would not respect them. The disjointedness of the system means that family physicians do not act as gate keepers of the healthcare system with patients visiting both, secondary care services and Emergency Departments directly. Some patients prefer Emergency Departments because it is easier to access, and they do not need to make an appointment or pay a fee. This affects the morale of family physicians as their patient list matters little in terms of referrals. The positive aspects recorded were that students viewed FM as offering guaranteed employment compared with secondary care.

## Action points: what medical schools internationally can do to encourage general practice as a career choice.

The consistent message from leaders of health care systems is that we need to produce more primary care physicians and fewer hospital specialists. This need has been heightened by the current COVID-19 pandemic. The following action points are practical steps that medical schools, supported by national governments can take to promote general practice:

1. Increase the time students spend in general practice during medical school.
2. Teach students about the theory and academic foundations that underpin general practice.
3. Present students with positive GP role models.
4. Allow interns to experience general practice in their first year or during a two-year foundation programme. Giving newly qualified doctors some experience of working in general practice will encourage them to consider this as a career option. When the foundation programme in the UK was created the plan was for all foundation programmes to include four months in general practice (GP Taskforce Final Report, 2013). This goal has not been reached. Currently only 50% of foundation programmes include general practice ( Dept. of Health, 2013).
5. Increase the capacity for speciality training/residency places for GP/Family Medicine programmes. In the UK, the number of places on GP training schemes was increased and in 2015 the fill rate was 89%; in 2016, after the first two rounds of processing applications it was 90%. There has now been significant increase in recruitment with the 2018 figures exceeding the 3250 places. This is a huge credit to the Primary Care Deans, Deaneries and HEE in the UK ( Cook, 2019) and shows what can be achieved when educators and the government work together.
6. Increase the length of training for general practice. For many years both the UK government and the RCGP have expressed a desire to increase the training scheme from three to four years in order to accommodate the expanded post-graduate curriculum. This has not happened.
7. Make it easier for doctors in training to switch to general practice. This could be done by giving them recognition of appropriate prior training, experience and expertise.
8. Do more to keep doctors who have trained as GPs to remain within the primary care workforce. This should include putting additional resources into the GP retainer and returner programmes.
9. Explore why at interview 25% of trainees to GP training are considered unsuitable (GP Taskforce Final Report, 2013).
10. Create financial equity of primary care and secondary care physicians. To make primary-care more attractive, the income of GPs has to achieve a degree of parity with the secondary sector.
11. Create innovative ways of reducing medical student debt, learning lessons from the Canadian model.
12. Ensure that medical schools and postgraduate training schemes produce doctors who represent their local populations who they are then happy to serve.
13. Those countries that do not have a national undergraduate curriculum could take the recent national undergraduate curriculum model from the UK ( Harding, Hawthorne and Rosenthal, 2018). There were key aspects of general practice that were taught by all schools, and this formed the basis of a national undergraduate curriculum published in 2018 as Teaching General Practice: Guiding Principles for Undergraduate General Practice Curricula in UK Medical Schools ( Harding, Hawthorne and Rosenthal, 2018).
14. Encourage GPs and hospital doctors to deliver undergraduate teaching in tandem not separately. Joint teaching ( Sandhu and Waddell, 2016) helps to build relationships and counter the poor opinion of general practice in medical schools.
15. Create and nurture student GP societies/clubs in all medical schools. The aim of these societies is to help fill the gaps in the formal medical school curricula and encourage students to think about a career in general practice.

Implementation of these action points will help to create a culture in which general practice is valued and attractive to new doctors.

## Conclusion

Globally governments are attempting to steer medical schools to produce more generalists and primary care physicians. By themselves medical schools cannot rebuild the primary care workforce. For instance, ‘The Shape of Training Report’ (2015) in the UK in its desire to produce more generalists, focused on the transition from medical school to the foundation programme and through to speciality training. Unfortunately, it did not incorporate the undergraduate curriculum in its scope. This then creates a divide between undergraduate teaching and postgraduate training. The reality is that if we are to produce graduates who are work-ready and have the competencies expected of an intern, medical education must be a continuum from undergraduate through to postgraduate education and training and continuous professional development.

Canada is an example of a country that has successfully encouraged its medical graduates to enter general practice. In the UK which historically had a large primary care workforce, some progress has been made in reversing the decline in the number of doctors entering general practice. This has been achieved by giving primary care greater prominence in the undergraduate curriculum and by expanding the number of places on GP training schemes. Meanwhile in the Middle East and Turkey where primary care started from a low base, much has been done to promote general practice as a career. In this article we have set out what else can be done across internationally to ensure that there are sufficient general practitioners to make primary care and the whole health care system effective.

## Take Home Messages

• Globally there is a chronic shortage of primary care physicians which is unsustainable.
• Medical Schools have a social responsibility to produce more primary care physicians.
• Undergraduate curriculum for primary care needs to be expanded.
• Students need more clinical exposure to primary care and positive GP role models.
• High quality primary care has a direct effect on the quality of patients’ lives.
• Governments and the medical profession need to value primary care.

## Notes On Contributors

**Prof Davinder Sandhu** was a Consultant Urological Surgeon at the University Hospitals of Leicester from 1992-2005 and subsequently became Prof of Medical Education and Postgraduate Dean, Severn Deanery, UK from 2005-2015. Recently he completed his 4 year term as Head of the School of Postgraduate Studies and Research, Royal College of Surgeons in Ireland - Bahrain. He is the holder of the Bruce Medal for education and training. ORCID ID: https://orcid.org/0000-0002-6344-1853

**Dr Andrew Blythe** is GP and Senior Teaching Fellow at the University of Bristol and Director of the MB ChB Programme (MB16) at Bristol Medical School. He has worked as a General Medical Practitioner (GP) in Bristol for 25 years. Previously he was Head of Teaching for Primary Care at the University of Bristol and is the first author of the undergraduate textbook *Essential Primary Care.*

**Prof Vijay Nayar** has been a GP for over 30 years and until recently was the Primary Care Dean in Health Educatin England, East of England. He is currently the National Clinical Director for the GP Induction and Refresher scheme. He is a Visiting Professor at Cranfield University and Honorary Senior Lecturer at the University of Hertfordshire.

## Appendices

Terminology: the authors wish to point out that internationally different terms are used for primary care physicians and in this paper the terms general practice, GPs, family medicine, community medicine are synonymous.
