# Supplementary material for: How can medical schools create the general practice workforce? An international perspective
Source: MedEdPublish (2016). 2020 Nov 27;9:267. [Version 1] doi: 10.15694/mep.2020.000267.1 (PMC10697486; doi:10.15694/mep.2020.000267.1)
Supplement: Peer review history

# Reviewer report: Reviewer response for version 1

**DOI:** 10.21956/mep.20187.r31223

**Published:** 2020-12-08

**Authors:** David Bruce

**Affiliations:** 1 None

This review has been migrated. The reviewer awarded 5 stars out of 5 This is a well written and informative paper that deals with a major problem in many countries - how we develop and train the required numbers of primary care physicians to serve population health needs. The paper sets the challenge to medical schools and postgraduate training authorities to ensure that they are producing a future medical workforce that meets the needs of patients.The evidence for the value of primary care in population heath is reviewed, and the UK report, By Choice and Not by Chance, which looks at the reasons for poor uptake in general practice as a career, is summarised. The authors then describe what is happening in the UK, USA, Canada, the Middle East and Turkey to promote primary care as an attractive career choice and expand the primary care medical workforce. I think the level of detail in this part of the paper is just right and there are lessons to be learned from each system. My only surprise in the UK section was that ScotGEM, new medical curriculum designed to develop doctors interested in a career as a generalist practitioner within NHS Scotland, was not discussed. The paper then proposes 15 practical steps that medical schools and postgraduate training authorities, supported by their governments, could take to promote general practice. I’m sure most will agree with all of the points made. What might be helpful would be to hear the authors views on which action points if any should be prioritised. An important paper and an enjoyable read. I’d recommend to all.

Reviewer Expertise:

NA

No decision status is available

# Reviewer report: Reviewer response for version 1

**DOI:** 10.21956/mep.20187.r31225

**Published:** 2020-12-01

**Authors:** Richard Hays

**Affiliations:** 1 James Cook University

This review has been migrated. The reviewer awarded 4 stars out of 5 This paper addresses an important question - how do medical schools produce the workforce that society needs? The issues mentioned are real, although an international perspective is difficult to achieve because of the variation is health systems, roles, status and incomes. Australia, New Zealand and East Asia have similar challenges and yet are not in this review, despite having a substantial proportion of the world’s population. My bias is that some Australian medical education initiatives are worth looking at - noticeably James Cook University and the rural clinical school movement, because although they are framed as rural, general/family practice is the most likely specialty involved. The list of potential improvement strategies is useful, although not all would work everywhere. James Cook University has achieved some success through combining several of theses strategies, because each on its own may be a relatively weak strategy. I worry about trying to place too much pressure on students and graduates e uses carrots are better than sticks. Achieving equivalent income and professional status may be important. Researching is very possible and indeed essential in primary care - almost the ultimate application in practice of many advances. However, many medical schools do not even believe that they have a responsibility to produce a particular kind of workforce, so work needs to be done within the profession and in universities. The article would be improved by including more of the works and considering what to do in the higher education sector.

Reviewer Expertise:

NA

No decision status is available

# Reviewer report: Reviewer response for version 1

**DOI:** 10.21956/mep.20187.r31224

**Published:** 2020-11-30

**Authors:** Trevor Gibbs

**Affiliations:** 1 AMEE

This review has been migrated. The reviewer awarded 5 stars out of 5 As a General Practitioner by training, I openly declare an eager interest in reading and reviewing this paper.After reading it however, I am unashamed in stating that it is a very thoughtful and well constructed paper that brings to the fore the decline in family medicine / general practice despite the evidence that states its importance in healthcare delivery and overall health improvement. The world appears at a crossroads in bolstering its healthcare services whilst trying to balance many other factors, explored within this paper. Much of the difficulty does appear to also rely upon the GP educational system (undergraduate and postgraduate), again explored within this paper. Not only does this paper explore the difficulties in the system but it provides some important answers and practical solutions for the way ahead.I highly recommend this paper to all those engaged within curriculum development, postgraduate GP training and healthcare management.

Reviewer Expertise:

NA

No decision status is available
